# Erector Spinae Plane Block Decreases Narcotic Requirements in Patients Undergoing Subcutaneous Implantable Cardioverter-defibrillator Placement Under Sedation

**DOI:** 10.19102/icrm.2024.15043

**Published:** 2024-04-15

**Authors:** Himani V. Bhatt, Jane Gui, Samit Ghia, Asad Mohammad, Hung-Mo Lin, Yuxia Ouyang, Dane Doctor, Bharat K. Kantharia, Davendra Mehta, Ali Shariat

**Affiliations:** 1 Department of Anesthesiology, Perioperative and Pain Management, Icahn School of Medicine at Mount Sinai, New York, NY, USA; 2 Department of Cardiovascular Surgery, Icahn School of Medicine at Mount Sinai, New York, NY, USA; 3 Department of Cardiology, Arrhythmia Institute, Icahn School of Medicine at Mount Sinai, New York, NY, USA; 4 Department of Anesthesiology, Yale School of Medicine, New Haven, CT, USA; 5 Department of Health Population Science and Policy, Icahn School of Medicine at Mount Sinai, New York, NY, USA

**Keywords:** Anesthesia, erector spinae plane block, opioids, subcutaneous implantable cardioverter-defibrillator implantation.

## Abstract

Providing adequate analgesia perioperatively during subcutaneous implantable cardioverter-defibrillator (S-ICD) implantation can be a challenge. The objective of our study was to assess the efficacy and safety of the erector spinae plane (ESP) block technique in providing analgesia and minimizing the risk of opioid use in high-risk patient populations. We enrolled consecutive patients >18 years of age undergoing S-ICD implantation from February 2020 to February 2022 at our center prospectively. Patients were randomly assigned to receive the ESP block or traditional wound infiltration. A total of 24 patients were enrolled, including 13 patients randomized to ESP block and 11 patients as controls who received only wound infiltration. The primary outcome assessed was the overall use of perioperative analgesic medications in the ESP block group versus the surgical wound infiltration group. A significant reduction in intraoperative fentanyl use was observed [median ([interquartile range]) in the ESP block group (0 [0–50] μg) compared to the wound infiltration block group (75 [50–100] μg) (*P* = .001). The overall postoperative day (POD) 0 fentanyl use was also significantly decreased (75 [50–100] μg) in the ESP block group compared to the surgical wound infiltration group (100 [87.5–150] μg) (*P* = .049). There was also a trend of decreased POD 0 oxycodone–acetaminophen use. Finally, the number of days to discharge was less in the ESP block group. These results indicate that ESP block is an innovative, safe, and effective technique that decreases intraoperative and postoperative opioid consumption and may be a useful adjunct pain-management technique in these high-risk patients. Larger studies are needed to further validate its use.

## Introduction

**Figure 1:**
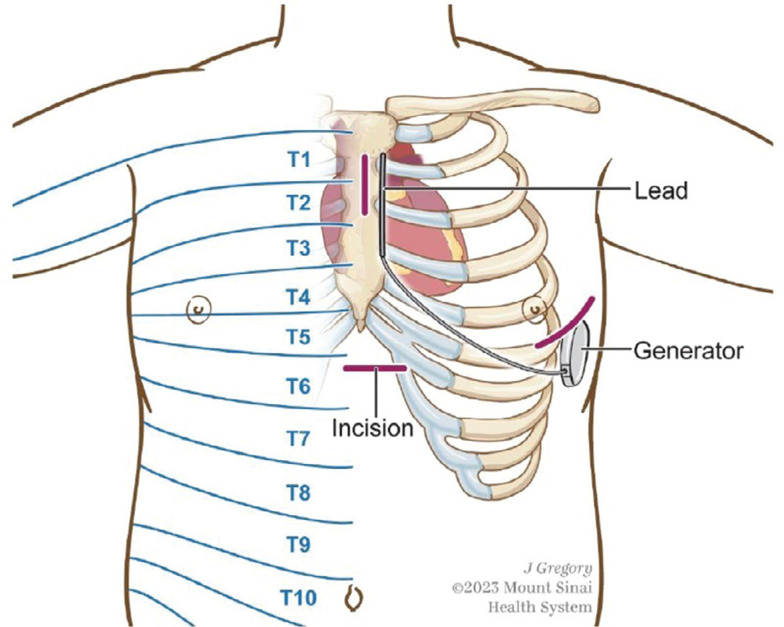
Dermatomal levels of incisions, tunneling site, and device.

As subcutaneous implantable cardioverter-defibrillators (S-ICDs) avoid cardiac and vascular complications, they are implanted as an alternative to transvenous implantable cardioverter-defibrillators (ICDs) to treat malignant ventricular arrhythmias.^1^ The procedure involves the placement of the generator subcutaneously in the left lateral chest wall and tunneling of the lead across and up the left parasternal border **(Figure 1)**. As this is a densely innervated region of the chest wall, analgesia can be a challenge. Traditionally, perioperative pain management for S-ICD placement is dependent upon wound infiltration with local anesthetics and the use of opioids. However, wound infiltration can result in unreliable efficacy due to the need to cover a large area of the anterior chest wall, the variable spread of the local anesthetic, and the limited duration of action. Furthermore, this patient population has multiple comorbidities, resulting in a greater risk for opioid-related side effects.

Regional techniques such as the erector spinae plane (ESP) block can provide good analgesia while attenuating the risk of opioids, especially in this patient population. The ESP block is proposed to provide a multidermatomal sensory block of the posterolateral and anterior thorax via anterior diffusion of local anesthetics to target the dorsal and ventral rami.^2–5^

In this study, we compared single-shot ESP block to surgical infiltration of local anesthesia for S-ICD placement. We hypothesized that because the ESP block may give coverage to both the parasternal and the inframammary tunneling sites during S-ICD placement, it would provide adequate analgesia during the operative and postoperative periods and therefore reduce the narcotic requirement in patients undergoing S-ICD implantation.

## Methods

### Study population and design

This was a single-center, prospective, randomized trial (ClinicalTrials.gov identifier: NCT04974762) in which patients undergoing S-ICD implantation from February 2020 to February 2022 at our institution were enrolled. The study was approved by our institutional review board (HS# 18-00362; GCO# 18-0776).

After providing written informed consent, 24 patients (convenience sample) >18 years of age undergoing S-ICD placement at a single-center tertiary care hospital were randomized using a method of sealed envelopes to receive either an ESP block or wound infiltration (control). Exclusion criteria included allergy to amide local anesthetics and infection at the site of injection. All procedures were performed with the administration of intravenous sedation with midazolam, fentanyl, and propofol infusion and supplemental oxygen. Patients refusing the block or patients requiring general anesthesia (GA) for procedural reasons were excluded. Neither the anesthesiologists performing the block nor the patients were blinded because there was no placebo block, and all blocks were performed prior to starting sedation. The anesthesiologist performing the block was part of the anesthesiology team. If the block was performed by the research team, the anesthesiologist performing the case was present during the performance of the block or had knowledge of the block and, therefore, was not blinded to the study group.

### Subcutaneous implantable cardioverter-defibrillator implantation technique

For S-ICD device implantation, patients were placed in a supine position on a surgical table, with the left arm in an abducted position on an armrest. There were three incisions made in total **(Figure 1)**, as follows: left parasternal, sub-xiphoid in the midsagittal line, and inframammary along the anterior axillary line. The device was situated in the anatomic space between the serratus anterior and the latissimus dorsi muscles at the T5–6 level. The distal tip of the electrode insertion tool was inserted at the xiphoid and tunneled laterally until the distal tip emerged in the device pocket. The electrode was then connected to the generator. The generator was fixed in the pocket with two separate sutures between the muscular fascia and the anchoring hole. The xiphoid incision site was also used to tunnel the lead cranially to reach the parasternal incision higher in the chest.

### Block and infiltration techniques

**Figure 2:**
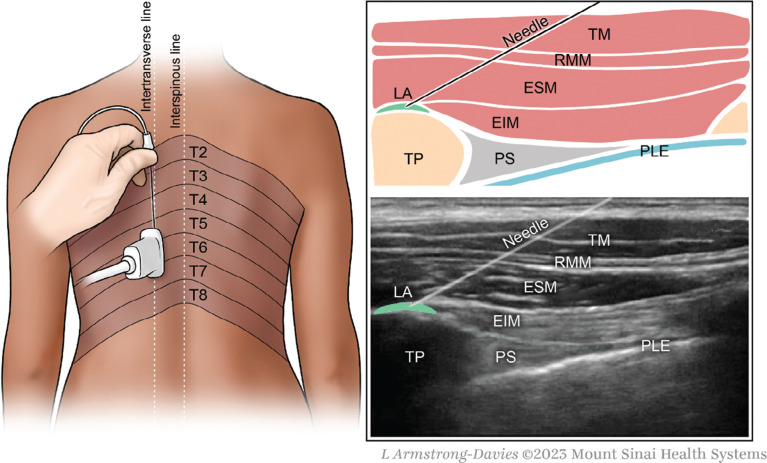
Erector spinae plane block at the T5–6 level using an in-plane approach visualizing local anesthetic spread between the trapezius muscle and external intercostal muscle. *Abbreviations:* EIM, external intercostal muscle; ESM, erector spinae muscle; ESP, erector spinae plane; LA, local anesthetic; PLE, pleura; PS, paravertebral space; RMM, rhomboid muscle; TM, trapezius muscle; TP, transverse process.

The appropriate level was determined by palpating the inferior angle of the scapula to identify the approximate level of the thoracic (T) 7 level. The T5–6 level could then be ascertained relative to the T7 level by scanning in the cranial direction with ultrasound visualization. A left-sided ESP block at the T5–6 level was performed under ultrasound guidance (Sonosite S-Nerve Ultrasound System fitted with an L38 £ 10- to 5-MHz transducer; Sonosite, Inc., Bothell, WA, USA) with a 22-gauge, 2-in. stimulating needle (Stimuplex^®^ A; B. Braun Medical Inc., Bethlehem, PA, USA) using an in-plane technique with the needle advancing in a cranial-to-caudal direction through the erector spinae muscle until the transverse process was contacted. A total of 20 mL of 0.25% bupivacaine was administered between the transverse process and the erector spinae muscle **(Figure 2)**. In the surgical infiltration group, 30 mL of 0.25% bupivacaine was injected into the implantation and tunneling sites before the incision. The patients in the ESP group did not receive any additional local anesthetics from the operating electrophysiologist team. Patients were administered an intravenous propofol infusion with intermittent boluses of intravenous midazolam and/or fentanyl as needed.

### Measured outcomes

The primary outcome was the overall use of perioperative analgesic medications in the ESP block group versus the surgical wound infiltration group. The administration of all pain medications, opioids, and non-opioids, including fentanyl, oxycodone–acetaminophen, hydromorphone, acetaminophen, ketamine, morphine, and tramadol, was documented. Fentanyl use was divided into two categories, induction and intraoperative use, to allow for comparison of fentanyl requirements during the procedure. Induction fentanyl use was defined as fentanyl use from anesthesia start to procedure start. Intraoperative fentanyl use was defined as any fentanyl administration thereafter until anesthesia was stopped. Secondary outcomes were visual analog scale (VAS) scores (0–10 points), intraoperative vital signs, total procedure time, total anesthesia time (block time was included), and length of stay in the intensive care unit (ICU). VAS scores were collected on the day they were measured.

### Statistical analysis

Descriptive data are reported as count (%), mean (± standard deviation), or median (interquartile range [IQR]) values, as appropriate. For group comparisons, Fisher’s exact test was used for categorical variables, the Student’s *t* test was used for normally distributed continuous variables, and the Kruskal–Wallis test was used for skewed continuous variables, as appropriate. Statistical analysis was performed using R version 4.1.1 in RStudio version 1.4.1717 (R Foundation for Statistical Computing, Vienna, Austria). All tests were two-sided, and statistical significance was defined as *P* < .05.

## Results

Among the study cohort of 24 patients (control vs. block group, 56.1 vs. 57.4 years old; 21% women) enrolled in the study, 13 were randomized to left ESP block and 11 were randomized to only wound infiltration (control). There were no significant differences between the two groups in terms of age; sex; body mass index; the American Society of Anesthesiologists score; ejection fraction; and comorbidities, including being on anticoagulation, atrial fibrillation, chronic obstructive pulmonary disease, smoking history, hypertension, diabetes mellitus, coronary artery disease, pulmonary hypertension, end-stage renal disease, and obstructive sleep apnea **(Table 1)**. None of the patients who were approached declined participation in the study, and none were excluded or lost to follow-up.

A significant reduction in intraoperative fentanyl use was observed: 0 (0–50) μg in the ESP block group versus 75 (50–100) μg in the wound infiltration block group (*P* = .001) **(Table 2)**. The overall postoperative day (POD) 0 fentanyl use was also significantly decreased: 75 (50–100) μg in the ESP block group compared to 100 (87.5–150) μg in the no-block group (*P* = .049).

There was a trend of decreased POD0 oxycodone–acetaminophen (5–325 mg) use, with a median of 0 tablets versus 1 tablet taken in the ESP block group versus control group, that did not reach statistical significance (*P* = .149). The day to discharge was shorter in the ESP block group at a median (IQR) of 1 (1–1) day instead of 1 (1–2) day(s) without the block (*P* = .038). No non-steroidal anti-inflammatory drugs were given. The use of other pain medications, including acetaminophen, ketamine, hydromorphone, morphine, and tramadol, did not differ between the two groups on POD0 or POD1.

No difference was noted in vital sign changes following incision, total procedural time, total anesthesia time that included the block time, or highest pain scores (including those recorded immediately postoperatively), on POD0 and POD1 **(Table 2)**. The median surgical time was 79 (73–89) min in the ESP block group and 83 (73–93.5) min in the wound infiltration group. Separately, the median pain scores were 0 (0–0) points in the ESP block group and 0 (0–1.5) points in the no-block group upon arrival at the postanesthesia care unit and 5 points on POD0 and POD1 for both groups, respectively. The median propofol use was 76.1 (53.0–101) mg in the ESP group and 66.5 (47.3–73.7) mg in the wound infiltration group, which was not statistically significantly different (*P* = .213). Similarly, there was no difference in the amount of midazolam administered. The mean length of stay in the ICU (wound infiltration vs. ESP block, 0 [0] vs. 0.182 [0.405] days) was also not different between the two groups (*P* = .116). There was no need to induce GA in any of the patients. No complications, including local anesthetic toxicity, hematoma, intrathecal injection, pneumothorax, or prolonged paresthesia, were observed.

## Discussion

In this prospective study, we demonstrate that the innovative ESP block technique can be easily and safely performed to provide analgesia during S-ICD placement under monitored anesthesia care (MAC). S-ICD insertion is a complex procedure often requiring GA with mechanical ventilation to provide an ideal surgical condition. However, in high-risk, cardiac-compromised patients who are undergoing S-ICD placement, avoiding GA can minimize hemodynamic instability and facilitate quick recovery. MAC is often safer for these high-risk patients. Nonetheless, deep sedation is often needed due to the pain-stimulating nature of the procedure, which includes parasternal tunneling and device insertion between muscle layers. The possibility of oversedation and transitioning to GA without a secured airway can, in turn, lead to increased mortality and morbidity.^6–8^

Previously, we reported transversus thoracic plane (TTP) and serratus anterior plane (SAP) blocks as safe and feasible analgesic adjuncts for S-ICD.^9^ There was a significant reduction in intraoperative fentanyl use, with a median of 45 versus 90 μg. Zhang et al. also showed that TTP and SAP blocks significantly reduced intraoperative dexmedetomidine and remifentanil use in patients undergoing S-ICD placement.^10^ Postoperatively, sufentanil use in the block group was half that in the local infiltration group; separately, ketorolac use in the block group was a quarter of that in the local infiltration group.^10^

The previous studies demonstrated the important role of truncal blocks in reducing intraoperative and postoperative pain medication use while performing S-ICD implantation safely under moderate sedation. However, there are greater risks of pneumothorax and internal mammary artery puncture with the TTP block due to the anatomical proximity of the fascia plane to the pleura and internal mammary artery.^11^ For a patient who has had an internal mammary coronary arterial bypass, the fascial plane injectate may not spread adequately, resulting in coverage of multiple dermatomes.^11^ On the contrary, ESP is a single trunk block that has the potential to cover the entire anterior thorax except for the sternum.^2^ This could provide analgesia for all incisional and tunneling sites **(Figure 1)**. The transverse process provides a safe landing zone for the needle tip to lower the risk of pneumothorax in case the needle tip cannot be well visualized during the block.^2^ ESP is a relatively easy block to perform with a steep learning curve. To the authors’ best knowledge, there have been no prospective studies comparing pain medication requirements between patients who received wound infiltration and ESP block for S-ICD placement under sedation. One retrospective chart review case series by Koller et al. showed that children who received parasternal and ESP blocks before extubation after S-ICD placement had a reduced narcotic requirement compared to those in the wound infiltration group.^12^

ESP blocks have been used successfully in many thoracic and cardiac surgeries. Studies have shown promising results. In video-assisted thoracic surgery, Ciftci et al. confirmed decreased total fentanyl consumption in the ESP group (176 vs. 717 μg) compared to the control group and significantly lower pain scores (passive and active) in the ESP group, especially in the first 8 h, via a prospective randomized study of 60 patients.^13^ Multiple studies showed decreased opioid use and speedier recovery in cardiac surgery patients who received ESP blocks.^14,15^ Krishna et al. showed that bilateral single-shot ESP blocks reduced the mechanical ventilation time from 102 to 63 min. Total opioid use was 231–935 μg. Most importantly, the time to ambulation was cut in half, from 62 to 36 h. Finally, the ICU stay length was 42 h instead of 70 h.^14^ Separately, Macaire et al. used bilateral ESP catheters in open cardiac surgery and demonstrated decreased total morphine consumption, postoperative nausea and vomiting, time to first mobilization, and pain scores at rest 1 month after surgery.^15^

Although these patients can also receive paravertebral or epidural blocks for intraoperative and postoperative pain control with the possibility of dense analgesia precluding GA or deep sedation, the loss of sympathetic tone can result in profound hypotension and bradycardia. Many of the patients requiring S-ICD have compromised cardiovascular systems and may develop hemodynamic instability, especially when combined with sedation. Furthermore, many patients are on anticoagulation; due to concerns for epidural hematoma, paravertebral and epidural blocks would require anticoagulation to be held ahead of time, which may not always be feasible.^16,17^ These factors have contributed to the limited use of such techniques in cardiac procedures. Novel truncal blocks, such as ESP, at the T5–6 level can provide analgesia to the T2–7 thorax by a local anesthetic spreading cranio-caudally and toward the paravertebral space.

The use of local anesthetic infiltration with sedation is safe and effective in most patients and is still the preferred method of management in many centers.^18^ Many electrophysiologists still use tumescent anesthesia, especially for larger areas of anesthetic coverage. Even though an ESP block is relatively safe and easy to perform, there is still a risk of pneumothorax and unintended epidural or intrathecal injection, and it may not reliably block the parasternal incision and tunneling site. In addition, ESP blocks are still considered an advanced block with a steep learning curve, often requiring an anesthesiology team that is well versed with the use of ultrasound and regional techniques. However, the benefit of decreasing even a small amount of opioid use intraoperatively and postoperatively can be beneficial in certain patient populations, such as the morbidly obese and patients with significant cardiac and pulmonary comorbidities, and the addition of an ESP block may provide larger benefits to these individuals than to the average patient.^19^ In addition, the ability to discharge patients early and safely with non-opioid or low-opioid pain management will most likely benefit this patient population.

The use of an ESP block did not increase the overall anesthesia time likely because it is a relatively easy block to perform and only one injection, whereas local infiltration requires injection of the entire tunneling sites, which is a large area. Furthermore, even under moderate sedation, patients likely move more due to the stimulation of the local injection at very densely innervated parasternal and inframammary sites. This can often prolong the procedural time and hence the overall anesthesia time.

The placement of an S-ICD can be painful, with a high risk of persistent opioid use (POU). As a matter of fact, Markman et al. showed that the incidence of POU in even opioid-naive patients can be as high as 12%, and therefore a multimodal pain approach, including truncal blocks, is recommended to reduce opioid use in these patients.^20^ In addition, recent studies have shown that even a small reduction in intraoperative opioid use can yield a significant decrease in postoperative complications, particularly in high-risk patients,^21,22^ although large studies are needed to determine this effect.

### Limitations

Our study has several limitations. Although prospective and randomized, our study was conducted at a single center. A well-powered multicenter study with a larger number of patients would be needed to determine outcomes and validate the use of this block as a standard of care for S-ICD placements. Neither the anesthesiologist nor the patient was blinded to the block, and hence an error of bias could have been introduced. Additionally, as shown in a few studies, a larger volume and/or higher concentration of bupivacaine may potentially provide a denser block and prolong the analgesic effect.^10,23,24^

## Conclusion

This study confirmed that ESP block is a safe and effective technique under MAC sedation in a multimodal analgesic regimen for S-ICD implantation. It led to a significant decrease in intraoperative and postoperative opioid consumption that may be of clinical importance in high-risk patients. Larger studies are needed to further investigate and validate the utility and, more importantly, the characteristics (i.e., volume and concentration) of ESP blocks as a standard for S-ICD implantation. In addition, larger studies would be required to further evaluate if the small decrease in opioid consumption can be applied to the larger population of patients for S-ICD implantation.

## Figures and Tables

**Table 1:** Demographic Characteristics

Characteristic	Wound Infiltration	ESP Block	*P* Value
	n = 11 (46%)	n = 13 (54%)	
	Mean (SD)/n (%)	Mean (SD)/n (%)	
Age, years	56.1 (10.8)	57.4 (11.4)	.778
Male sex	8 (72.7%)	11 (84.6%)	.63
BMI, kg/m^2^	26.5 (4.96)	29.8 (4.48)	.109
ASA score	3.55 (0.522)	3.31 (0.480)	.249
EF	32.1 (13.6)	43.3 (19.5)	.114
HTN	6 (54.5%)	10 (76.9%)	.39
CAD	7 (63.6%)	8 (61.5%)	1
DAPT	5 (45.5%)	3 (23.1%)	.39
DM	4 (36.4%)	1 (7.7%)	.142
AC	2 (18.2%)	0 (0%)	.199
Smoker	2 (18.2%)	0 (0%)	.199
ESRD	2 (18.2%)	0 (0%)	.199
Pulm HTN	1 (9.1%)	0 (0%)	.458
COPD	0 (0%)	1 (7.7%)	1
OSA	0 (0%)	0 (0%)	NA
AF	0 (0%)	0 (0%)	NA

*Abbreviations:* AC, anticoagulation; AF, atrial fibrillation; ASA score, American Society of Anesthesiology Physical Status Classification score; BMI, body mass index; CAD, coronary artery disease; COPD, chronic obstructive pulmonary disease; DAPT, dual antiplatelet therapy; DM, diabetes mellitus; EF, left ventricular ejection fraction; ESRD, end-stage renal disease; HTN, hypertension; NA, not applicable; OSA, obstructive sleep apnea; Pulm HTN, pulmonary hypertension.

**Table 2:** Clinical Outcomes

Variables	Wound Infiltration	ESP Block	Kruskal–Wallis Test *P* Value
	n = 11 (50%)	n = 13 (50%)	
	Median [Q1, Q3]	Median [Q1, Q3]	
Intraoperative			
Induction fentanyl, μg	0 [0, 50.0]	50.0 [50.0, 50.0]	.0463*
Intraoperative fentanyl, μg	75.0 [50.0, 100]	0 [0, 50.0]	<.001*
Propofol, mg	66.5 [47.3, 73.7]	76.1 [53.0, 101]	.213
Midazolam, mg	2 [2, 2]	2 [2, 2]	.9952
Postoperative day 0			
Fentanyl, μg	100 [87.5, 150]	75.0 [50.0, 100]	.0493*
Oxycodone–acetaminophen 5–325 mg (no. of tablets)	1 [0, 1.5]	0 [0, 1]	.149
Hydromorphone IV, mg	0 [0, 0]	0 [0, 0]	.358
Postoperative day 1			
Oxycodone–acetaminophen 5–325 mg (no. of tablets)	0 [0, 1]	1.5 [0.25, 2]	.211
Hydromorphone IV, mg	0 [0, 0]	0 [0, 0]	.317
Pain score (0–10), points			
Immediately in PACU	0 [0, 1.5]	0 [0, 0]	.790
Highest on POD0	5 [3, 6.5]	[0, 8]	.813
Highest on POD1	5 [3, 6]	5 [1, 6.5]	.908
Procedure time			
Total surgery time, min	83.0 [73.0, 93.5]	79.0 [73.0, 89.0]	.685
Total anesthesia time, min	156 [131, 169]	150 [139, 164]	.622
Days until discharge	1 [1, 2]	1 [1, 1]	.038*

*Abbreviations:* PACU, postanesthesia care unit; POD0, postoperative day zero; POD1, postoperative day one. *P values are statistically significant.
